# Advanced System-on-Chip Field-Programmable-Gate-Array-Powered Data Acquisition System for Pixel Detectors

**DOI:** 10.3390/s24010218

**Published:** 2023-12-30

**Authors:** Jorge Jiménez-Sánchez, Pedro Blanco-Carmona, José María Hinojo-Montero, Francisco Rogelio Palomo, Rafael Luis Millán, Fernando Muñoz-Chavero

**Affiliations:** Department of Electronic Engineering, University of Sevilla, 41092 Sevilla, Spain; pblanco@us.es (P.B.-C.); jhinojo@us.es (J.M.H.-M.); fpalomo@us.es (F.R.P.); rmillan@us.es (R.L.M.); fmunoz@us.es (F.M.-C.)

**Keywords:** data acquisition systems, edge computing, pixel detectors, particle tracking, hybrid pixel, read-out chip, system-on-chip, FPGA, embedded microprocessor, telescope

## Abstract

Particle detector systems require data acquisition systems (DAQs) as their back-end. This paper presents a new edge-computing DAQ that is capable of handling multiple pixel detectors simultaneously and was designed for particle-tracking experiments. The system was designed for the ROC4SENS readout chip, but its control logic can be adapted for other pixel detectors. The DAQ was based on a system-on-chip FPGA (SoC FPGA), which includes an embedded microprocessor running a fully functional Linux system. An application using a client–server architecture was developed to facilitate remote control and data visualization. The comprehensive DAQ is very compact, thus reducing the typical hardware load in particle tracking experiments, especially during the obligatory characterization of particle telescopes.

## 1. Introduction

High-energy particle detectors in physics are critical sensors for the detection, identification, kinematic tracking, and energy measurement of elementary particles produced in collider experiments [1].

**Figure 1:**
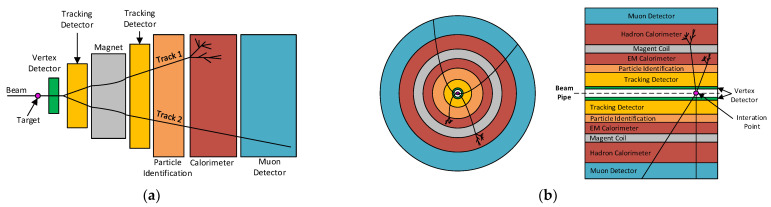
Configurations of detector components: (**a**) side view in fixed-target setup and (**b**) beam orientations in collider experiments.

In general, to achieve complete detection of all particle parameters, detectors are usually constructed in layers (planar layers in the case of fixed targets (Figure 1a) and shell layers in the case of colliders (Figure 1b)), with each layer dedicated to a specific task.

This work focused on the design and implementation of a data acquisition system (DAQ) for tracking detectors. The information obtained from the detectors using the DAQ could be used to reconstruct the trajectory, determine the momentum (via magnetic fields) and kinematic properties, and identify the particles. For this purpose, the tracking detectors were placed in the first layers, usually near the collision point and in front of and/or behind a magnetic field.

For particle tracking, silicon-based pixel array detectors are currently the most widely used due to their high granularity and low cost [2]. These detectors provide high spatial resolution (on the micron scale), ensuring precise particle trajectory identification. Additionally, their efficiency in high-radiation environments and adaptability to high-density electronic readout systems have bolstered their widespread adoption.

**Figure 2:**
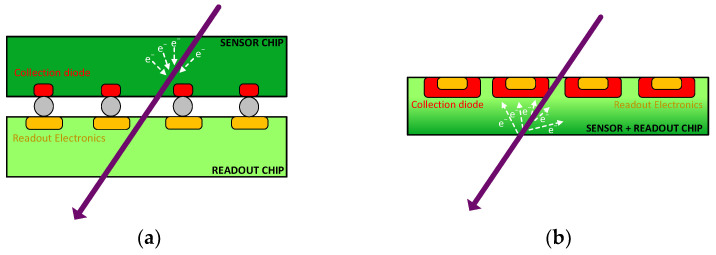
Comparison between hybrid sensors (**a**) and monolithic sensors (**b**).

There are two types of silicon-based pixel detectors: hybrid sensors and monolithic sensors. The hybrid sensor (see Figure 2a) consists of a chip with a sensitive volume (detector) connected to another chip containing the control and readout electronics. In contrast, monolithic sensors (see Figure 2b) contain both the sensors and the readout electronics in the same silicon block [3]. Both types of sensors are undergoing significant development and are being used in different applications [4]. Monolithic sensors [5,6] are used in applications where mass reduction and a low material budget are the most limiting requirements. An example of their use and performance can be found in the STAR [7] experiment or in the work published by the RD50 collaboration [8]. Likewise, hybrid particle detectors, although they began being used in environments that required high rates and radiation, such as the LHC [9], in recent years, are being applied in fields such as photon science, medical imaging, or electron microscopy, where the budget limitation is not a limiting factor and where the versatility and power of these systems have allowed for achieving important breakthroughs [10,11].

The readout electronics in a particle detector are typically complex systems that integrate analog front-ends and high-speed digital circuits. On the one hand, the analog front end is responsible for converting the charge generated in the sensor by the particle impact into an electrical signal that can be processed. On the other hand, digital circuits are used to obtain the measurement results (high-speed signals) and to configure the operation of the particle detector (low-speed signals) using parameters such as bias and reference voltage or to select a particular pixel sensor to drive the output. Some detectors deliver the analog signal directly, necessitating external digitization for processing, while others feature onboard digitization for streamlined external handling.

**Figure 3:**
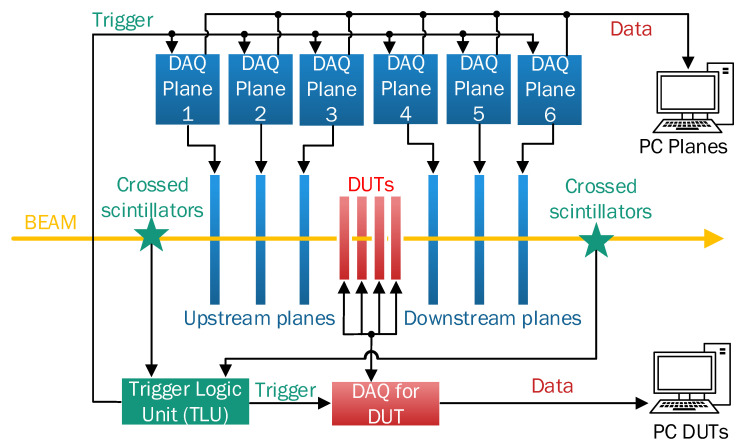
Beam telescope configuration for testing new particle sensors.

Each new particle-tracking sensor must be tested in a beam telescope (Figure 3) before installation in a collider. The beam telescope is a simplified version of a complete tracking detector, consisting of reference pixel detectors and trigger scintillators, with the detector (device under test (DUT)) planes interleaved between the reference planes. The trigger signals come from the scintillators placed before and after the tracking block. A trigger logic unit (TLU) processes the trigger signal and sends it to the DAQ systems, as well as to various adapter boards that may be required to connect the tracking planes to their respective DAQ systems. This enables a comparison of the tracking information from the reference pixel detector planes with the data obtained from the detectors under test (DUTs) to calibrate their performance and quality.

It is important to note that the DAQ acts as an interface between the read-out electronics and the PC that processes the digitized data. The primary objective is to retrieve data from one or more particle detectors for real-time processing and, potentially, storage for further analysis. In addition, it is essential that the acquisition process is synchronized with the trigger signal provided by the TLU to capture the particle passage event; otherwise, the information generated will not be correlated with the measurement made.

The goal of this study was to improve the previous data acquisition system (DAQ) for the ROC4SENS chip used in particle tracking and to enable the management of multiple readout chips for information collection in telescope experiments. The ROC4SENS integrated circuit was developed at the Paul Scherrer Institute (PSI) [12] and corresponds to a hybrid particle detector. It provides a direct analog output that must be converted to the digital domain.

The implemented DAQ system is based on a system-on-chip (SoC) FPGA, which integrates a programmable logic and a microprocessor on the same package. The use of an SoC FPGA simplifies the communication interface; provides a fully functional operating system (OS); and adds parallel processing capability for communication, control, and data acquisition from the detector through the logic fabric. In addition, the use of this system allows for the addition of other pre-processing capabilities to the system or communication with databases in the cloud. The proposed system is also capable of managing up to four chips simultaneously. This feature simplifies the experimental setup by reducing the number of DAQs to be deployed during a test. In addition, having a single DAQ improves the synchronization of the detectors, as the clock source is the same for all of them, and will also allow individual corrections to be made to the trigger event received for each detector.

The new DAQ system implemented offers significant advantages over the previous data acquisition system of the ROC4SENS chip, based on an Altera Cyclone III FPGA. First, it can successfully reach the maximum frequency of the ROC4SENS chip (160 MHz), enabling a fast-reading mode where the value of a pixel is obtained in each clock cycle, achieving the maximum reading speed. To take advantage of the SoC’s capabilities, we used DMA transfer techniques to move the acquired data into the RAM, freeing the microprocessor from other tasks and not limiting the speed of operation. Moreover, the communication interface with the user was upgraded from a USB 2.0 interface to a Gigabit Ethernet interface. The integration of a microprocessor-based SoC running a fully functional Linux operating system allows for the adaptation and direct execution of the CERN EUDAQ software [13] on the system.

This new DAQ implementation represents a significant advancement compared with several older data acquisition systems that utilized earlier-generation FPGA-based systems. These systems include the DAQ for the EUDET Pixel Telescope (EUDRB) controlled by an ALTERA Cyclone II FPGA device [14,15]; the DAQ for the Alice monolithic sensor developed by the A Large Ion Collider Experiment collaboration, which relies on a Xilinx Kintex-7 FPGA [16]; and the DAQ for ROC4SENS itself, which is based on an Altera Cyclone III FPGA [17].

The implemented system aligns with the prevailing trend of DAQs employing SoC FPGA technologies. Some examples include the autonomous measurement system for the silicon beam tracker and test benches in the BM@N (Baryonic Matter at Nuclotron) experiment, which consists of a control system based on a Xilinx Zynq-7020 SoC within a MicroZed system on module (SOM) [18]. The high-intensity proton synchrotron DAQ, which accelerates protons in the J-PARC Main Ring (MR) [19], is based on an Altera Cyclone V SX SoC. The Caribou DAQ, which supports multiple detectors, is based on a Xilinx Zynq-7000 SoC [20]. The SPIDR4 DAQ [21], which is used for the Timepix4 chip, is based on the Xilinx Zynq-7000 family SoC. Lastly, the Beam Instrumentation PiXeL (BIPXL) MPSoC Readout System [22] is used for the Timepix3 hybrid pixel detectors. Additionally, the KamLAND2 experiment is upgrading its DAQ system to incorporate RFSoC technology for high-speed sampling and data acquisition [23].

This article is structured as follows: a description of the readout chip used and its current DAQ is given in Section 2. The proposed implemented DAQ system and its experimental measurements are presented in Section 3 and Section 4, respectively. Finally, some conclusions are drawn in Section 5.

## 2. Sensor ROC4SENS

For the design of the proposed acquisition system, we opted for the versatile ROC4SENS readout chip developed by PSI. This read-out chip was implemented with the main objective of reducing the overall cost and development time of new hybrid particle detection sensors. Along with the read-out chip, the PSI team developed a readout system called the digital test board (DTB) [24] to manage and read out a single ROC4SENS chip. In addition, a board to generate the supply and reference voltages for the chip (DTB adapter) and a board to support the chip (PCB support) were implemented.

This section describes each of the elements that make up the complete ROC4SENS system.

### 2.1. Read-Out Chip ROC4SENS

The ROC4SENS integrated circuit is a versatile readout chip originally designed for the Compact Muon Solenoid (CMS) experiment [25,26]. The readout architecture of ROC4SENS is highly adaptable, facilitating its use with different arrays of overlapping pixel detectors.

Some of the key features of this chip include the following [27]:

- Analog pulse measurement from particle impacts.
- Implementation without pixel zero suppression to allow for studies of charge accumulation on the sensors.
- Radiation-resistant design.
- Fast front-end amplifier optimized for LHC conditions.
- External control for flexibility in readout.
- Pulse injection mechanism for calibration.
- Compatibility with readout hardware and wafer test systems already in use for readout chips used in CMS pixels.
- Minimal on-chip programming to reduce recalibration requirements.

The chip was designed using a standard 250 nm CMOS technology, which is known for its radiation tolerance, meeting the requirements for the CMS upgrade [28]. The total chip area was 7.8 mm × 9.8 mm. Importantly, the ROC4SENS pixel array consisted of 155 columns and 160 rows, allowing up to 24,800 pixels to be soldered. The connection was made via a staggered bump link pattern with a pitch of 50 µm. 

**Figure 4:**
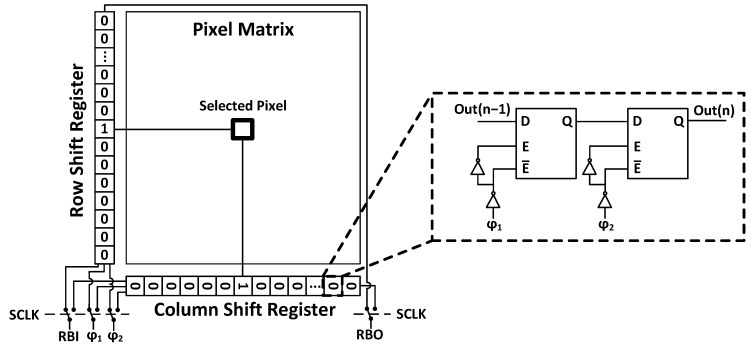
ROC4SENS matrix.

Two separate shift registers (SRs) were used for pixel selection and located just outside the pixel array (see Figure 4). 

The pixels are read sequentially to avoid summation of the stored charges in the pixels during readout. External control allows for readout and calibration of any desired number and order of pixels in both SRs.

**Figure 5:**
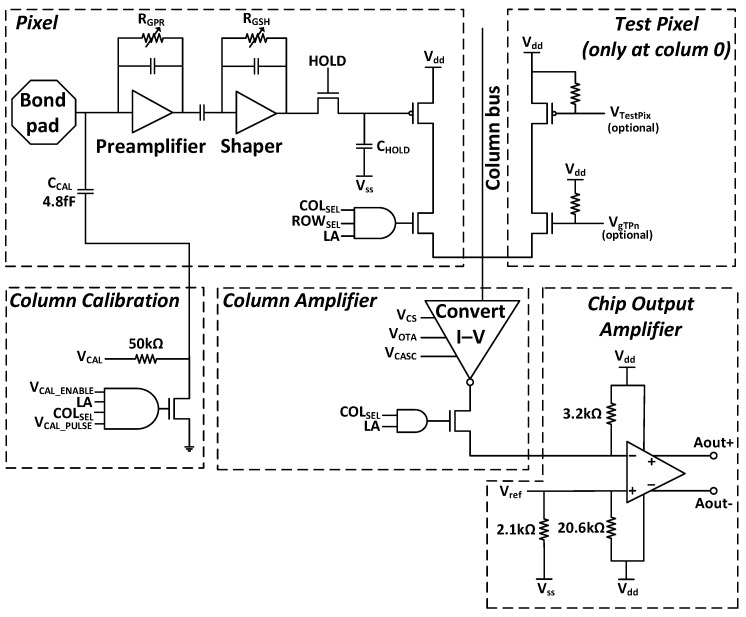
The ROC4SENS read-out chain.

Signal transfer occurs at the column level, with all pixels in a column connected to a common column bus and I–V converter. All column amplifiers are, in turn, connected to a single differential output amplifier. Each pixel comprises a read-out chain with a pre-amplifier and shaper for rapid pulse shaping in LHC conditions, storing charge using a sample-and-hold circuit. Figure 5 shows a schematic of the read chain available on the ROC4SENS chip.

The operation of the chip is controlled by a series of control signals listed in Table 1.

To work with the pixel, after receiving the data acquisition trigger signal from the TLU, the HOLD signal is clear to enable the analog memory capacitor C_Hold_ and store the hit. To select the pixel to read, we used the configuration shift registers (column and row), via the RBI, φ1, φ2, and SCLK signals. The analog memory capacitor charge is converted to voltage and transferred to the differential output amplifier by a selection logic. A reference voltage is used in the differential output amplifier to adjust the signal range [29]. 

For electrical testing, the ROC4SENS chip has a calibration mode that works by injecting a current pulse into the existing C_CAL_ capacitor in each pixel. All signals controlling the calibration mechanism are global. The voltage is provided by VCAL and the VCAL_PULSE signal allows for controlling the timing of the calibration pulse. The calibration pulse is generated at the periphery, separately for each column. 

Emulating particle impact energy is a very useful tool, as it avoids the need to use a particle source or pulsed laser too often during the development phase of the data acquisition system. The calibration mode can be run without any sensors connected to the ROC4SENS readout chip.

### 2.2. PCB Support and DTB Adapter

The ROC4SENS chip is directly bonded to a carrier board (PCB support card). This PCB support is connected to the DTB adapter carrier board, which supplies analog and digital supply voltages and reference signals using two MAX584 [30] digital-to-analog converters (DACs). Biasing of the high voltage detector is also provided by the DTB adapter. The digital test board drives the adapter board via a ribbon cable.

### 2.3. Digital Test Board (DTB)

To test the ROC4SENS chip, a data acquisition system called the digital test board (DTB) was created. 

The DTB is based on an Altera Cyclone III FPGA that includes 128 MB of DDR2 RAM and supports a MicroSD card. The FPGA can be programmed using USB or JTAG interfaces. For data communication, the system incorporates a USB 2.0. The acquisition block is composed of an ADC that operates at 100 Msamples/s. It also includes connectors for interconnection with the TLU [31].

## 3. Proposed Data Acquisition System

The proposed data acquisition system (DAQ) was specifically designed for use in particle tracking detectors in telescope experiments. To achieve this, a DAQ was designed to allow for the control and simultaneous acquisition of up to four integrated sensors. Incorrect synchronization will cause a drift in the reconstructed particle trajectory. Therefore, having one acquisition system for each chip complicates data synchronization, as all DAQs must share the same clock reference, and the connection scheme must be carefully planned to minimize mismatches and loss of synchronization. The architecture fits into an edge-computing design philosophy.

Furthermore, the system was integrated into a SoC FPGA, which includes a microprocessor to simplify the communication interface. The operating system running on the microprocessor embedded in the SoC enables direct sensor interaction and potentially supports additional pre-processing capabilities.

These features allow the system to stay up-to-date with current trends in SoC implementation. By using the ROC4SENS chip, our system inherits certain functionalities from the DTB test system in terms of chip control, with specific updates:

- Simplification in assembly: unlike the current DTB system, which necessitates a DAQ for each sensor, the proposed system supports all four sensors within a single DAQ.
- Enhanced synchronization with the TLU interface, offering the flexibility to use a trigger channel for each chip (for individual operation) or a common channel for all.
- Achievement of the ROC4SENS chip’s maximum operating frequency (160 MHz).
- Improved the data transfer speed when communicating with the user, moving from a USB 2.0 interface on the DTB to a Gigabit Ethernet interface.
- Use of analog-to-digital converters with a rate of 160 MSPS to transform the chip’s differential analog output (in contrast to the DTB system’s rate of 100 MSPS).
- Data transfer via DMA from the FPGA logic fabric to the microprocessor on the SoC.

**Figure 6:**
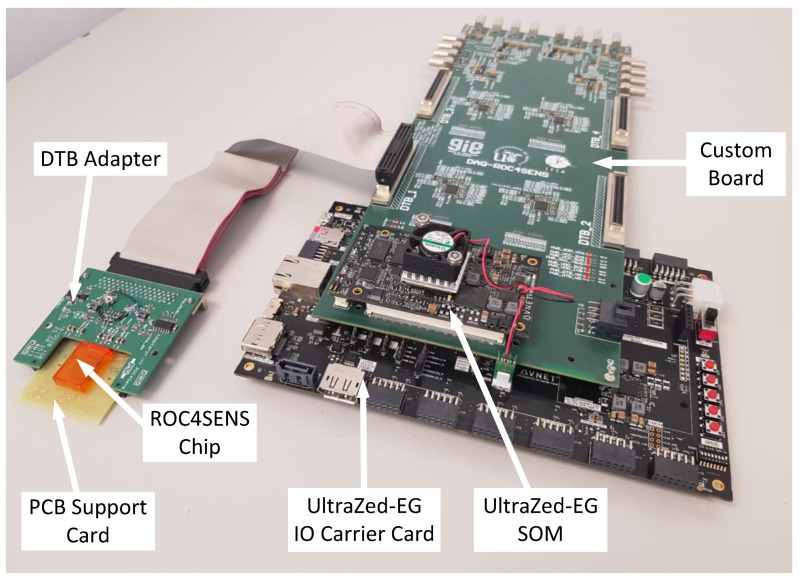
Implemented data acquisition system.

The implemented system is based on three boards: an UltraZed-EG SOM board from AVNET [32], the associated UltraZed-EG IO Carrier Card [33], and a custom PCB designed for the application. In addition, the chips are connected to the custom PCB via a ribbon cable. We retained both the PCB support and the DTB adapter (to generate chip supply and reference voltages) from the previous system. Moreover, a dedicated power supply board was designed to allow for remote system power management. Figure 6 shows an image of the developed system.

The off-the-shelf commercial system, which combines the UltraZed-EG SOM with the UltraZed-EG IO Carrier Card, initially lacked an adequate number of I/O pins for connecting to the chips. To overcome this limitation, a custom PCB was introduced into the system. As a result, all the general-purpose I/O pins of the UltraZed-EG SOM are available on the custom PCB. The custom PCB also contains the ADCs to convert the chip’s analog output and the connections to the TLU for system triggering. 

The UltraZed-EG SOM incorporates a Xilinx Zynq UltraScale+ MPSoC system-on-chip (SoC), specifically the XCZU3EG-1SFVA625 device [34]. This SoC integrates the following:

1. Programmable logic block (71k LUTs, 154k logic cells, and 7.6 Mb total block RAM).
2. Quad Arm Cortex-A53 (up to 1.5 GHz) main processor.
3. Dual Arm Cortex-R5F (up to 600 MHz) for real-time processing.
4. Arm Mali-400 MP2 (up to 667 MHz) graphics processor.

This SoC is connected to the rest of the system via three high-density connectors that distribute the appropriate signals to the UltraZed-EG IO Carrier Card and the custom PCB. The functions provided by these connectors include 156 I/O pins for the signaling required by the ROC4SENS, triggers (TLU) and ADC (located on the custom PCB), and connections to integrate the most common communication interfaces and peripherals located on the UltraZed-EG IO Carrier Card.

The UltraZed-EG IO Carrier Card is compatible with the UltraZed-EG SOM. It connects via three high-density connectors to the custom PCB to which the SoC (UltraZed-EG SOM) is connected. The card provides the following:

1. USB 2.0/3.0.
2. USB-UART.
3. Gigabit Ethernet
4. SD card storage interfaces.
5. Power rails for the UltraZed-EG SOM, including a 12 V main input voltage.

The system is initiated with software commands sent from the SoC’s microprocessor to its programmable logic (FPGA), which then communicates with the chips via the custom board. When a particle event is detected by the TLU, it sends an activation signal to the custom PCB, capturing data from the chips. The custom PCB’s ADCs convert the data, which is then transferred to the SoC’s programmable logic. Finally, the data is written to the SoC’s memory through DMA for analysis in the microprocessor’s operating system.

The following subsections describe the hardware implementation and the firmware and software of the developed system.

### 3.1. Hardware

**Figure 7:**
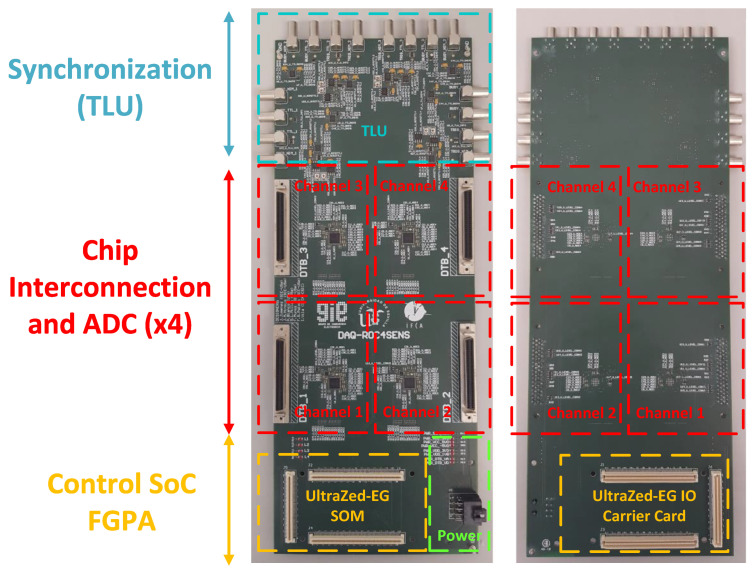
Custom board PCB (top PCB on the left and bottom PCB on the right).

The custom PCB (Figure 7) is a 10-layer PCB designed specifically for the system. It contains the necessary hardware to enable connectivity between the programmable logic within the UltraZed-EG SOM, the UltraZed-EG IO Carrier Card, the ROC4SENS chips (via the DTB adapter), and the TLU for the system trigger.

This board is organized to simplify connections in the experimental setup. At the top of the board are the TLU synchronization interfaces (four interfaces, working individually for each ROC4SENS). In the middle, organized by channel, are the interfaces with the DTB adapter card and the ADCs (four interfaces and four ADCs, one for each ROC4SENS). At the bottom of the board are the connections to the UltraZed-EG SOM (top of the board) and the UltraZed-EG IO Carrier Card (bottom of the board). Finally, in the bottom right-hand corner is the interface to the power supply board.

#### 3.1.1. UltraZed Interconnection Block

This block directly connects the UltraZed SOM and the UltraZed-EG IO Carrier Card for communication interfaces (USB, Ethernet), storage (via SD card), and the required power supply for the UltraZed SOM. In addition, the general-purpose I/O pins of the UltraZed SOM are available on the custom PCB for connection to both the ROC4SENS detectors and other board components.

At the bottom of the board are three high-density connectors for linking to the UltraZed-EG IO Carrier Card, and at the top of the board are another three high-density connectors for linking to the UltraZed-EG SOM.

#### 3.1.2. Adapter Card Interconnection Block

This block contains the signals necessary to control the ROC4SENS, the chip output (differential analog), and the I2C connections for communication with the two DACs of the DTB adapter. It also provides the supply voltages for the DTB adapter. The connection is made using a SCSI type connector and a ribbon cable.

For safety reasons, the high voltage required to bias the detector is not routed through the custom board but is added via the same ribbon cable from a separate power supply.

#### 3.1.3. ADCs Block

The UltraZed SOM only supports digital signals, and thus, it is necessary to convert the ROC4SENS chips’ differential analog output to digital. For this purpose, there are four ADCs on the custom board (one for each interface).

The chosen ADC is the 12C170 [35]. It has a resolution of 12 bits and the output is a single parallel CMOS. This ADC requires no configuration and initiates conversion via an enable signal. Both the enable signal and the clock come from the programmable logic implemented in the UltraZed SOM.

#### 3.1.4. TLU Triggers Block

This block implements the necessary hardware to interact with the TLU to use the telescope trigger. The system offers the flexibility to utilize either a single trigger for all four interfaces or individual triggers for each chip, allowing for individual testing. Therefore, an interface to the TLU is implemented for each ROC4SENS interface.

As the TLU can work with both TTL and NIM formats, interfaces for both formats were implemented (four TTL format TLU interfaces and four NIM format interfaces). The use of LEMO-type connectors ensures compatibility with TLUs employed in CMS and various other telescope setups.

#### 3.1.5. Power Supply Block

In this block, we have a Molex header for connection to the power supply board, which provides all the voltages required for the custom PCB and the DTB adapter cards for the various interfaces. This, in turn, provides the reference and power voltages for the ROC4SENS chips.

### 3.2. Programmable Logic

The logic for controlling the ROC4SENS chips, data acquisition, and communication with the TLU for telescope triggering was implemented in the fabric logic of the SoC.

In addition, a driver for the operating system was developed (details are given in the following sections of the article). This driver was implemented in a PetaLinux distribution and runs on the Quad Arm Cortex-A53 of the SoC. The driver sends commands to the programmable logic implemented to control the ROC4SENS chips and to visualize the acquired data.

**Figure 8:**
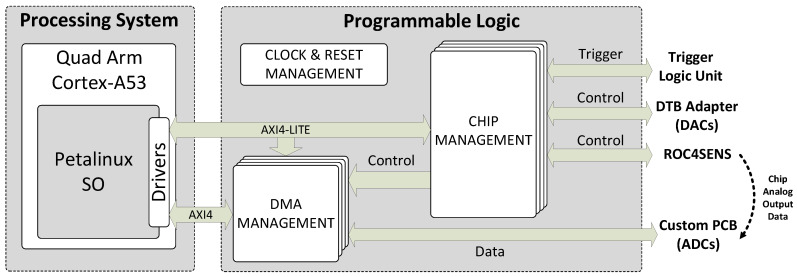
Block diagram of the implemented logic.

Figure 8 illustrates the programmable logic in the SoC, which was developed using VHDL and Verilog via Xilinx Vivado 2020.2. It comprises clock and reset management, chip management (four instances, one for each ROC4SENS interface), and DMA management (four instances, one for each ADC interface). The general operation is as follows: Commands from the OS interact with chip management blocks, which interface with ROC4SENS chips, DTB adapter cards (via I2C for generating reference voltages), and TLU for event trigger synchronization. After the trigger signal from the TLU data is captured, the ADCs on the custom board convert the chips’ analog output to digital. The DMA management block collects digital data and writes it directly to memory for subsequent OS analysis.

#### 3.2.1. Clock and Reset Management Block

The clock and reset management block is responsible for generating the different system clocks and the resets associated with each of the clock domains. There are three clock domains in the system: a 40 MHz clock as the main system clock, corresponding to the frequency of the CERN LHC; 160 MHz for the control signals of the ROC4SENS chips; and 100 MHz for operating system management and DMA. 

This block distributes the clock and reset signals to the rest of the system blocks and also provides the clock signal for the ADCs on the custom board. 

For the ADCs, there is a mechanism for disabling the clock when the ADC is not in use. This mechanism is controlled by the user via control commands.

#### 3.2.2. Chip Management Block

The chip management block consists of an AXI4-Lite interface and the ROC4SENS control block (responsible for signaling and managing the ROC4SENS chip), DAC control (communicating with the DTB adapter DACs to generate the chip supply and reference voltages), and trigger control (responsible for communicating with the system trigger received via the interface with the TLU).

**Figure 9:**
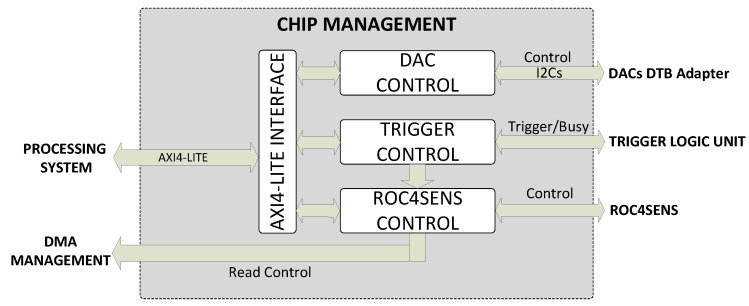
Chip management block diagram.

A more detailed diagram of this block is shown in Figure 9.

The operation of the chip management block starts with the reception of commands sent by the operating system implemented in the processor. The AXI4-Lite interface transforms the AXI4-Lite type commands into the native interface. Then, in the same interface block, a change from the 100 MHz clock domain (the frequency at which the AXI interfaces of the processor are configured) to the main system clock domain of 40 MHz is performed via a FIFO. Finally, depending on the type of command, it is passed to the appropriate block (ROC4SENS control, DAC control, or trigger control).

If the commands are for chip control, they are sent to the ROC4SENS control block. This block is responsible for managing the chip signals for shock capture, matrix readout, or calibration.

The block can store several commands in a FIFO memory. When the start command is sent from the operating system, it starts to execute each of the stored commands.

The ROC4SENS control block, on the one hand, receives the trigger signal from the trigger control block (which communicates with the TLU through the interfaces implemented in the custom board) to acquire the shocks and, on the other hand, sends the valid data signal to the DMA management block when reading the matrix to acquire the data from the ADC (integrated into the custom board).

The implemented commands are shown in Table 2.

**Figure 10:**
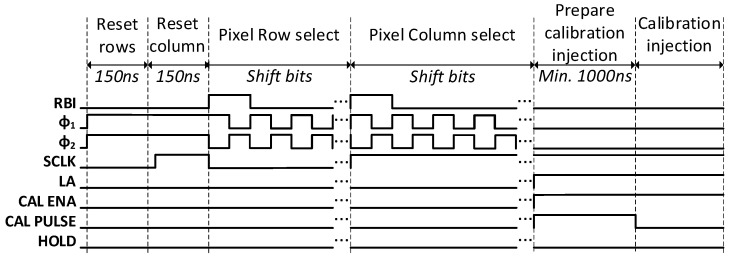
Timing with the control signals of the chip for the calibration of the pixel.

Figure 10 shows an operational example of the timing of the pixel calibration control signals.

As the chip operates at a maximum frequency of 160 MHz, the ROC4SENS control block switches from the main system clock domain (40 MHz) to the clock used by the ROC4SENS control signals (160 MHz).

The ROC4SENS control block is based on a Verilog design made by PSI for the DTB system [36] and was adapted to our system by making several modifications to adapt it to the new architecture.

On the one hand, if the communication is intended for the generation of chip supply/reference voltages, the commands received are transferred to the DAC control block. This block implements an I2C master for communication with the DACs in the DTB adapter.

Finally, if the commands are intended to control the system trigger, they are passed to the trigger control block. This block is responsible for configuring the communication interfaces with the trigger and for sending the capture signal to the ROC4SENS control block.

The trigger control block has the ability to share a single interface for the whole system (for all four chips), an individual interface for each chip, or various combinations (as the configuration is done independently for each chip). You can also configure the signal format to be used (TTL or NIM).

#### 3.2.3. DMA Management Block

**Figure 11:**
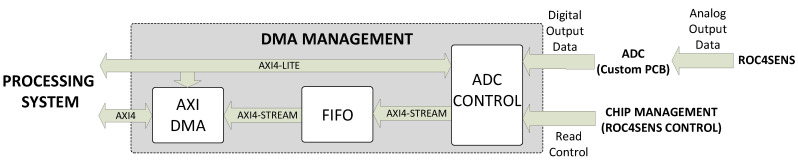
DMA management block diagram.

The DMA management block consists of an ADC control block, a FIFO, and the DMA block. A more detailed diagram of this block is shown in Figure 11.

The operation of the DMA management block by the ADC control block starts with the activation of the ADC by the user through commands sent via the AXI4-Lite interface (the ADC clock is activated when the ADC is activated).

When the matrix (or a single pixel) is read, it receives control signals from the chip management block (specifically the ROC4SENS control block contained in the previous one), which controls and manages the chip. 

The matrix can be read in rows or columns. The chip management block starts by sending a row (column) read start signal, a signal for each pixel shift within the row (column), and a row (column) read end signal.

In the case of a single-pixel read, only the pixel read start signal and the pixel read end signal are received.

For both matrix and single-pixel readouts, the ADC control block has two modes of operation:

- Continuous mode: the block captures all samples received from the ADC from the time the read start signal (row/column or single pixel) is received until the read end signal (row/column or single pixel) is received.
- Single mode: The block takes only one sample for each pixel of each row/column or one sample for a single pixel read. In the case of a matrix read, the capture is determined by the row (column) read start signal for the first pixel and the pixel offset signal within the row (column) for the remaining pixels. In the case of a single-pixel read, the capture is determined only by the pixel read start signal. In both cases, a capture offset can be configured.

In both modes, the ADC control block captures the data, organizes it into bursts, and converts it into a data stream in the AXI4 stream format.

The ADC control block has several registers that can be configured by sending commands from the processor. The configurations that can be made are the capture mode, the burst size, ADC delay cycles, and the capture offset (for continuous mode only).

The data bursts are then stored in the FIFO memory, which is responsible for resolving the clock domain conversion (the data source operates at 160 MHz, while the new clock domain operates at 100 MHz).

The FIFO memory sends the data to the AXI DMA block to write the data directly to memory. Once received, the data is available for display by the operating system. 

In order to have a modular and independent system for each ROC4SENS chip and to simplify the software for data handling, a DMA (together with a FIFO) is used for each sensor.

### 3.3. Software

The operating system is a Petalinux distribution [37] compiled with tools provided by Xilinx. This tool uses the bitstream file and the hardware description file generated in Vivado 2020.2 after the firmware implementation. To automate the OS compilation process and the OS implementation, a script was created to perform the OS compilation. The OS integrates everything needed to communicate with the outside world via Ethernet (SSH). 

To communicate with the programmable logic, a software driver was implemented in C/C++ to control and configure the different blocks, initialize the DMAs, and retrieve the memory data.

**Figure 12:**
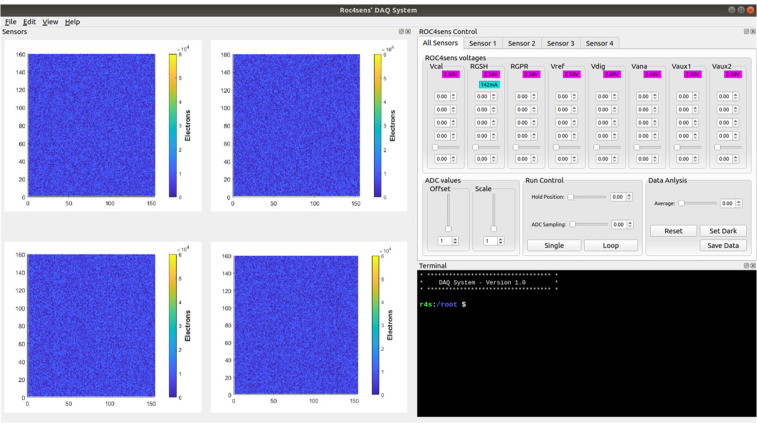
Graphical interface in QT.

Finally, a graphical interface (see Figure 12) was developed in QT (made with QT Creator 4.9.1 based on QT 5.12.3) that allows the user to interact with the proposed DAQ from any remote host (or locally from the Petalinux operating system). This remote software uses remote procedure calls (RPCs) to establish Ethernet communication between the GUI and the DAQ, allowing for remote data acquisition.

This system, in addition to being able to configure the sensors and their polarization voltages and display the results of the matrix, allows the results to be saved in “comma-separated values” (csv) format for further processing.

## 4. Results

To validate the functionality of the implemented data acquisition system, a calibration test was performed using the ROC4SENS chip. This readout chip has the ability to perform calibration procedures. A global calibration signal injects a charge at the input of the preamplifier via a capacitor implemented in each pixel. Its analog behavior is therefore equivalent to a sensor signal. It is, therefore, not necessary to connect a sensor to the ROC4SENS readout chip to inject the calibration pulse.

**Figure 13:**
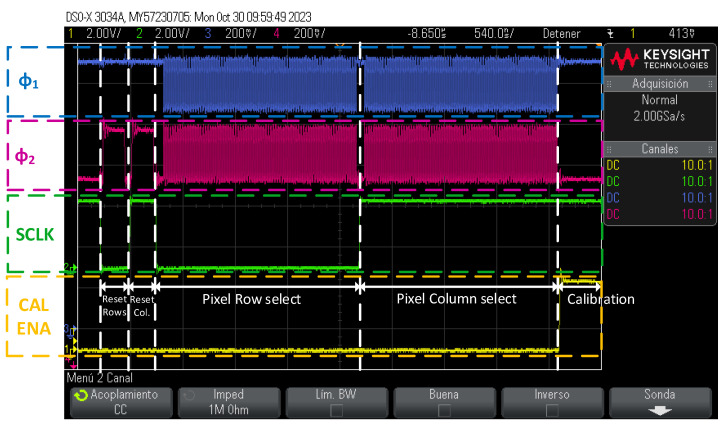
Pixel calibration sequence using oscilloscope capture.

First, a single-pixel calibration was performed to analyze the behavior in the pixels. This was done by applying a global calibration voltage to all pixels (VCAL PULSE signal) and charging the internal calibration capacitor at each pixel. The oscilloscope was used to check that the control signals and the timing of these signals were correct, as shown in Figure 13. A sequence of commands was executed to first reset the row and column shift registers and then select a single pixel (selecting its corresponding row and column). Finally, a calibration injection was performed on the selected pixel.

The calibration pulse at the pixel was determined by the charging time of this capacitor and the voltage we applied externally. We maintained a pulse length that was long enough to fully charge the capacitor (minimum 1000 ns) and varied the VCAL-PULSE voltage to record the results of the different calibration pulses.

For this first test, we used the continuous mode implemented in the DAQ, where all the samples received from the ADC were continuously captured from the moment the start reading signal was received until the end reading signal was received (in this case from the individual pixel). 

For all tests, a digital voltage of Vdig = 2.2 V was used for the chip; an analog voltage of Vana = 2.0 V; a Vref = 230 mV was used as the output amplifier reference for the chip; and Rgsg = 600 mV and Rgpr = 600 mV were configured as feedback voltages for the shaper and preamplifier, respectively.

**Figure 14:**
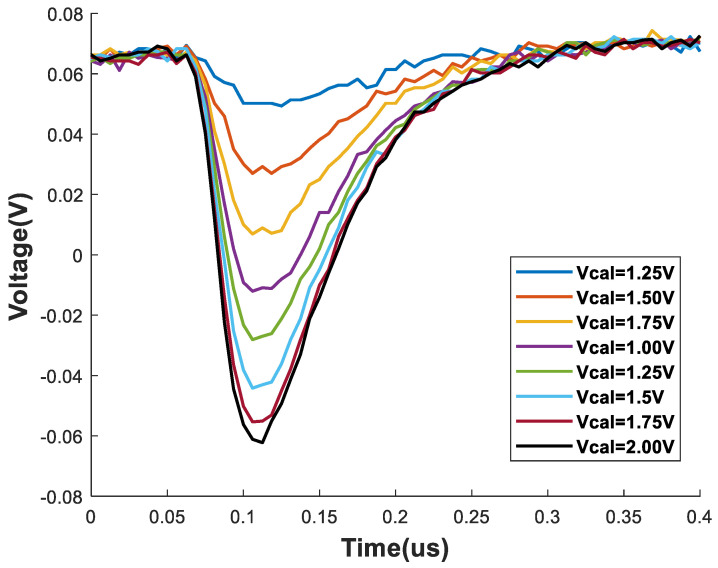
Calibration test results for a single pixel using different calibration voltages and the continuous mode of the DAQ. Biasing voltages were Vdig = 2.2 V, Vana = 2.0 V, Vref = 230 mV, Rgsg = 600 mV, and Rgpr = 600 mV.

The calibration results on the selected pixel are shown in Figure 14. The calibration voltage was varied from 250 mV to 2 V, which corresponds to impacts that deposit charges of 7500 to 60,000 electrons.

Once the calibration was obtained for one pixel, a complete calibration of the matrix was performed in each of the four available channels of the implemented DAQ. 

**Figure 15:**
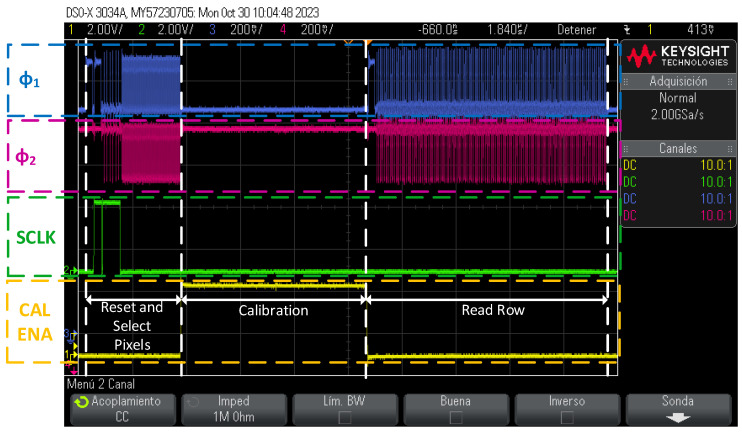
Reading and calibrating row sequence using oscilloscope capture.

For each of the channels, a sequence of commands has been executed to read and calibrate the matrix row by row. The procedure started by resetting the row and column shift registers. Then, the system was positioned on the first row and the pixels to be calibrated were selected (the calibration was performed on the whole column, and thus, the columns to be calibrated were selected). Finally, the selected pixel columns were calibrated and then the entire row was read. The same procedure was then carried out for the next row until the matrix was complete. The procedure with the control signals is shown in Figure 15.

**Figure 16:**
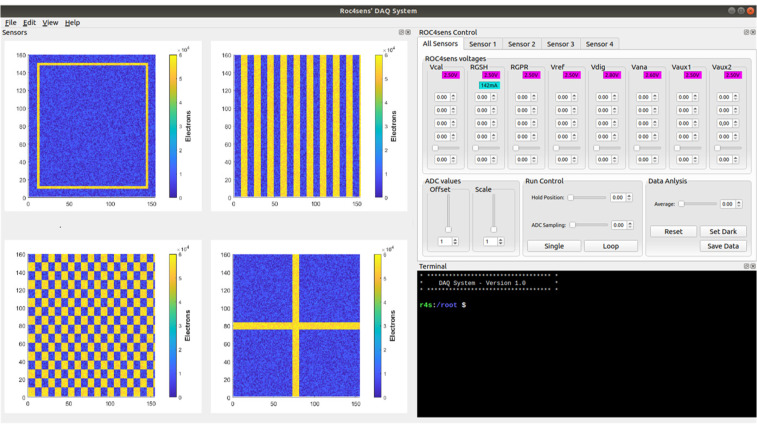
Full readout and calibration on each of the four channels.

Finally, by running different calibration configurations on each of the DAQ channels and using the DAQ single mode, only one sample was taken for each pixel in each row. The implemented graphical interface was used for this test and the result is shown in Figure 16.

The calibration voltage used in all calibrations was 2 V, which corresponded to an impact that deposited a charge of 60,000 electrons. For the matrix readout, using the fast readout mode on the ROC4SENS chip, the maximum frame rate approached 1.5 kHz.

## 5. Conclusions

In summary, this work successfully met the proposed objectives of designing and implementing a data acquisition system (DAQ) capable of controlling and obtaining information from multiple tracking detectors in telescope experiments.

The system demonstrated the ability to acquire signals from four integrated sensors simultaneously, which simplifies the experimental setup and facilitates the synchronization of events and the correlation of data from different detectors to determine the trajectory of particles. 

The implemented DAQ system is based on a system on a chip (SoC) that integrates an FPGA and microprocessor. Having an FPGA SoC enables the edge-computing paradigm, which allows for processing close to the source of information (in our case the detector) with the aim of sending the processed information to higher levels, thus reducing the amount of raw data. It also simplifies the communication interface, provides a fully functional operating system, and adds parallel processing capability for communication, control, and data acquisition from the detector through programmable logic.

Specifically, the DAQ system was designed to acquire and control four ROC4SENS hybrid sensors developed at the Paul Scherrer Institute (PSI). This new system has significant advantages over the previous ROC4SENS chip data acquisition system based on FPGA technology. These advantages include simplified assembly by supporting all four sensors in a single DAQ and the ability to operate at the maximum frequency of the ROC4SENS chip, achieving a maximum readout speed of 160 MHz. Analog-to-digital converters at 160 MSPS are used to achieve this maximum readout speed, and DMA transfer techniques are used to accelerate communication between the FPGA and the microprocessor in the SoC. In addition, the user communication interface was upgraded from USB 2.0 to a Gigabit Ethernet interface.

The inclusion of an SoC with a microprocessor running a fully functional Linux operating system allows the EUDAQ software used at CERN to be adapted and run directly on the system, allowing high-level processing and the ability to remotely upload data to the CERN database.

Moreover, the implemented system improves versatility by facilitating adaptation to different reader chips. In the case of detectors with analog output, adapting the system would mean replacing the controller within the programmable logic. Furthermore, in terms of hardware, the adaptation would require the modification of the adapter card (DTB adapter for ROC4SENS) in order to connect the corresponding chip to the custom board PCB of the proposed system. In the case of detectors with digital outputs [38,39,40], the hardware adaptations would go beyond the changes mentioned above, as they would also involve the elimination of the ADCs and the direct connection of the digital outputs to the FPGA SoC. In terms of software, the changes would be minimal and would involve adapting the configuration parameters for the new chip and visualizing the results for different pixel array sizes.

Finally, given the capacity of the microprocessor, the data could be pre-processed to eliminate false hits or other analyses, which would reduce the amount of data and simplify the analysis of the results. Additionally, a web server could be included for results analysis, control, and visualization.

## Figures and Tables

**Table 1:** Control signals for the ROC4SENS chip.

Signal	Type	Description
φ1	LVDS	Clock for the selected shift register
φ2	LVDS	Clock for the selected shift register
RBI	LVDS	Read bit-in shift register
RBO	LVDS	Read bit-out shift register
LA	LVDS	Connects pixels shift register output
HOLD	LVDS	Connects the sample-and-hold capacitor
SCLK	SE	Selects shift register for columns or rows
VCAL-ENABLE	SE	Enable calibration
VCAL-PULSE	SE	Injection of the calibration pulse

**Table 2:** ROC4SENS commands.

Command	Description
Start/Stop	Execute or stop commands stored in the FIFO
Resetx	Resets the shift register for row selection
Resety	Resets the shift register for the column selection
Loadx	Pixel row selection
Loady	Pixel column selection
Measure	Single pixel reading
ReadLine	Read entire row
FirstLine	Positioned on the first line
NextLine	Positioned on the next line
ReadColumn	Full-column reading
FirstColumn	Positioned in the first column
NextColumn	Positioned in the next column
ExtTrigger	Activates the storage of the impact after the trigger
Calib	Calibration

## Data Availability

Data are contained within the article..

## Abbreviations

The following abbreviations are used in this manuscript:

ADC	Analog-to-digital converter
CERN	European Organization for Nuclear Research
CMS	Compact Muon Solenoid
CSV	Comma-separated values
DAC	Digital-to-analog converter
DAQ	Data acquisition system
DTB	Digital test board
DUT	Device under test
FPGA	Field programmable gate array
LHC	Large hadron collider
OS	Operating system
PSI	Paul Scherrer Institute
RCP	Remote procedure call
ROC	Read-out chip
SoC	System-on-chip
SoM	System-on-module
SR	Shift register
TLU	Trigger logic unit
